# Stressing the Ubiquitin-Proteasome System without 20S Proteolytic Inhibition Selectively Kills Cervical Cancer Cells

**DOI:** 10.1371/journal.pone.0023888

**Published:** 2011-08-31

**Authors:** Ravi K. Anchoori, Saeed R. Khan, Thanasak Sueblinvong, Alicia Felthauser, Yoshie Iizuka, Riccardo Gavioli, Federica Destro, Rachel Isaksson Vogel, Shiwen Peng, Richard B. S. Roden, Martina Bazzaro

**Affiliations:** 1 Department of Pathology, Johns Hopkins University, Baltimore, Maryland, United States of America; 2 Department of Oncology, Johns Hopkins University, Baltimore, Maryland, United States of America; 3 Department of Biochemistry and Molecular Biology, University of Ferrara, Ferrara, Italy; 4 FDA-CDER Division of Product Quality Research, Silver Spring, Maryland, United States of America; 5 Masonic Cancer Center and Department of Obstetrics, Gynecology and Women's Heath, University of Minnesota Twin Cities, Minneapolis, Minnesota, United States of America; 6 Department of Gynecology and Obstetrics, Johns Hopkins University, Baltimore, Maryland, United States of America; University of Pennsylvania School of Medicine, United States of America

## Abstract

Cervical cancer cells exhibit an increased requirement for ubiquitin-dependent protein degradation associated with an elevated metabolic turnover rate, and for specific signaling pathways, notably HPV E6-targeted degradation of p53 and PDZ proteins. Natural compounds with antioxidant properties including flavonoids and triterpenoids hold promise as anticancer agents by interfering with ubiquitin-dependent protein degradation. An increasing body of evidence indicates that their α-β unsaturated carbonyl system is the molecular determinant for inhibition of ubiquitin-mediated protein degradation up-stream of the catalytic sites of the 20S proteasome. Herein we report the identification and characterization of a new class of chalcone-based, potent and cell permeable chemical inhibitors of ubiquitin-dependent protein degradation, and a lead compound RAMB1. RAMB1 inhibits ubiquitin-dependent protein degradation without compromising the catalytic activities of the 20S proteasome, a mechanism distinct from that of Bortezomib. Treatment of cervical cancer cells with RAMB1 triggers unfolded protein responses, including aggresome formation and Hsp90 stabilization, and increases p53 steady state levels. RAMB1 treatment results in activation of lysosomal-dependent degradation pathways as a mechanism to compensate for increasing levels of poly-ubiquitin enriched toxic aggregates. Importantly, RAMB1 synergistically triggers cell death of cervical cancer cells when combined with the lysosome inhibitor Chloroquine.

## Introduction

Ubiquitin-dependent protein degradation via the ubiquitin-proteasome system (UPS) is crucial for the regulation of many cellular processes including cell cycle progression, differentiation and apoptosis in both normal and cancer cells [1]. Aberrant expression of components of the UPS system including ubiquitin-ligases, de-ubiquitinating enzymes and proteasomes has been reported in several cancer settings including cervical cancer [1], [2], [3], suggesting that in order to sustain their higher levels of metabolic activity cancer cells rely more heavily upon the proper function of the UPS as compared to their normal counterpart [4], [5], [6], [7]. Thus, molecules capable of interfering with ubiquitin-dependent protein degradation, including Bortezomib, show anticancer activity [5]. Human Papillomavirus (HPV) is the primary cause of cervical cancer and responsible for 5% of all cancers worldwide [8]. While HPV vaccines can be an effective preventive measure against cervical cancer, there are currently no virus-specific therapies for it, and the efficacy of standard surgical and chemo/radiotherapies is limited for advanced disease [9]. Expression of two viral oncogenes, E6 and E7, is necessary for the induction and maintenance of the transformed phenotype [10]. The E6 oncoprotein exerts its oncogenic activity by binding to the E3 ubiquitin ligase E6-AP and redirects its activity towards p53 and other tumor suppressor proteins for their rapid ubiquitin-mediated proteasomal degradation [11], [12], [13]. This reduces the level of this key cellular cell cycle regulator without its mutation. Therefore, we hypothesized that stabilization of p53 via preventing its ubiquitin-mediated degradation will have therapeutic potential for cervical cancer and possibly for other cancers wild-type for p53.

Natural compounds of the flavonoid and triterpenoids families including curcumin, Celastrol, green tea polyphenols and chalcones have shown promise as antineoplastic agents in a variety of cancer settings including cervical [14], colon [15], [16], oesophageal [17], pancreatic [18] and prostate [19], [20], [21] cancer, linked to pro-apoptotic properties as associated with proteasomal inhibition. We have recently shown that chalcone-derivatives containing single aminoacid substitutions in their structure act as proteasome inhibitors and that the nature of the aminoacidic portion determines their selectivity toward the different catalytic activities of the 20S proteasome [14]. However other findings suggest that chalcone molecules might contain within their α- unsaturated carbonyl system the molecular determinant for inhibition of ubiquitin-mediated protein degradation upstream of the 20S proteasome [22], [23], [24], [25].

We report for the first time that a series of chalcone-derivatives lacking aminoacidic components, here termed RAMBs, are ubiquitin-proteasome system (UPS)-stressors via inhibition of ubiquitin-mediated protein degradation upstream of the 20S proteasomal catalytic activites. Specifically, our RAMBs compounds are capable of selective killing of cervical cancer cells via accumulation of poly-ubiquitinated protein followed by triggering of unfolded protein responses including aggresome formation and Hsp90 stabilization. Further, this accumulation of poly-ubiquitinated proteins is accompanied by a compensatory activation of lysosome-dependent protein degradation, stabilization of p53, the destabilization of cyclin D1 and the onset of apoptosis. Our findings suggest that treatment RAMB compound, possibly combined with the lysosome inhibitor Chloroquine, has promise as new avenue for the treatment of cervical cancer.

## Materials and Methods

### Cell culture

Cervical cancer cell lines HeLa, SiHa, CaSki and ME180, were obtained from American Type Culture Collection (Manassas, VA) and cultured in DMEM supplemented with 10% fetal bovine serum, 100 IU/mL penicillin, and 100 µg/mL streptomycin at 5% CO_2_. Keratinocytes were obtained from Invitrogen (Carlsbad, CA) and cultured in defined Keratinocyte-SFM.

### Cell viability assay

Cell viability was determined by 2,3-bis[2-methoxy-4-nitro- 5-sulfophenyl]-2H-tetrazolium-5-carboxanilide inner salt (XTT) assay (Roche Diagnostics GmbH, Mannheim, Germany). Cells seeded at the concentration of 1,000 per well in 100 µL medium in 96-well plate were treated with chalcone-based derivatives at specified concentrations. After the indicated periods, the cells were incubated according to the manufacturer's protocol with the XTT labeling mixture for 4 hours. Formazan dye was quantified using a spectrophotometric plate reader to measure the absorbance at 450 nm (ELISA reader 190; Molecular Devices, Sunnyvale, CA). All experiments were done in triplicate.

### Determination of apoptotic cells by flow cytometry

Induction of apoptosis was determined by Annexin-V/7-AAD staining. Annexin-V/7-AAD staining was done using Annexin V-PE Apoptosis Detection Kit I (BD Pharmingen, San Diego, CA) according to manufacturer's protocol. Briefly, 1×10^5^ cells were re-suspended in Binding Buffer, 5 µl of Annexin V-PE and 5 µl of 7-AAD were then added into the cells which were then incubated at room temperature for 15 minutes, and analyzed by flow cytometry on a Becton Dickinson FACSCalibur. Data analysis was done with CellQuest software (Becton Dickinson Immunocytometry System, Mountain View, CA).

### Antibodies and Western Blot Analysis

Total cellular protein (10–20 µg) from each sample was separated by SDS-PAGE, transferred to PVDF membranes and subjected to Western blot analysis. Antibodies for Western Blot analysis were obtained by the following commercial sources: anti-ubiquitin (Santa Cruz Biotechnology, Santa Cruz, CA), anti-p53 clone DO-1 (Calbiochem, Gibbstown, NJ) anti-PARP (BD Pharmingen, San Diego, California), anti-GAPDH (Sigma, St. Louis, MO), peroxidase-linked anti-mouse Immunoglobulin G (Amersham, Piscataway, NJ) and utilized at the concentration recommended by the manufacturer. Anti-ubiquitin and anti-vimentin antibodies for immunofluorescence analysis were obtained from Santa Cruz Biotechnology (Santa Cruz, CA). Texas red-labeled goat anti-mouse Immunoglobulin G and Fluorescein-labeled horse anti-rabbit Immunoglobulin G were obtained from Molecular Probes (Carlsbad, CA), and Vector Laboratories (Burlingame, CA) respectively and used at the concentration recommended by the manufacturer.

### Cellular morphology and immuno-fluorescence analyses

A Nikon Eclipse TE 2000E inverted microscope was used for the imaging of cellular morphology with phase contrast and immunofluorescence. For analysis of ubiquitin and vimentin sub-cellular localization, cultures of HeLa cells were grown as described in Lab-Tek II chambered coverglass (Nalge Nunc International, Rochester, NY). At the indicated times, cells were fixed and permeabilized with methanol and incubated with the indicated primary antibodies. Fluorescent secondary antibodies were used to visualize protein localization and nuclear DNA visualized by 4,6-diamidino-2-phenylindole (DAPI) staining. Mounted samples were viewed under a Nikon Eclipse TE 2000E inverted microscope and images captured with Spot 3.5.8 acquisition software (Diagnostic Instruments, Sterling Heights, MI).

### Measurement of Proteasomal activity in 20S Purified Proteasomes

Cells (5×10^8^) were washed in cold PBS and resuspended in buffer containing 50 mM TRIS-HCl (pH 7.5), 5 mM MgCl_2_, 1 mM DTT (Sigma), 2 mM ATP and 250 mM sucrose. Glass beads equivalent to the volume of the cell suspension were added, and the mixture was vortexed for 1 min at 4°C. Beads and cell debris were removed by 5 min centrifugation at 1,000 g, followed by 20 min centrifugation at 10,000 *g* [27]. Lysates were cleared by ultracentrifugation for 1 hr at 100,000 *g*, and supernatants were further ultracentrifuged for 5 hr at 100,000 *g*. Proteasome-containing pellets were resuspended in 0.5 ml of homogenization buffer [50 mM TRIS-HCl (pH 7.5), 100 mM KCl, 15% glycerol]. Protein concentration was determined using the BCA protocol (Pierce, Rockford, IL). Fluorogenic substrates Suc-LLVY-AMC, Boc-LRR-AMC and Ac-YVAD-AMC were used to measure chymotryptic-like, tryptic-like and caspase-like activities, respectively. Semipurified proteasomes (10 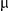
l), pretreated or not with inhibitors for 30 min at 37°C, were assayed at 37°C for 45 min using the different peptide substrates in a buffer containing 50 mM TRIS-HCl (pH 7.5), 5 mM MgCl_2_ and 1 mM DTT (final volume 100 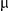
l). The reaction was quenched with 1 ml 1% SDS and fluorescence determined by fluorimeter (Perkin-Elmer, Beaconsfield, UK) with excitation at 380 nm and emission at 440 nm [18]. Data are expressed as the percent inhibition relative to untreated proteasomal preparations.

### Measurement of Proteasomal activity in 26S Proteasomes in living cells

Exponentially growing cells (1×10^6^) were plated in 60 mm dishes and either mock treated or treated with different concentration RAMB1 over a period of 4 hours. Proteasomal activity in cell lysates (NP-40 lysis buffer: 0.1% NP-40, 50 mM Tris-HCl pH 7.5, 150 mM NaCl, 5% glycerol and 1 mM DTT) was determined by measuring residual luminescence activity following addition of the Suc-LLVY-Glo™ substrate (Promega, Madison, WI) specific for the chymotrypsin-like activity of the proteasome according to the manufacturer's recommendations.

### Assay of 4XUbiquitin-Luciferase degron

The 4Xubiquitin–luciferase fusion construct designated Ub-FL and the control plasmid designed CMV-FL were kindly provided by Dr. David Piwnica-Worms (Washington University, St. Louis, MO) [26]. Sub-confluent cultures of HeLa cells were transfected with plasmids DNA by using Lipofectamine 2000 reagent (Life Technologies, Carlsbad, CA). FL and Ub-FL transfected HeLa cells were seeded at 50,000 cells/well in 24-well plates or 200,000 cells/well in 6-wells plate 24 hours post transfection and incubated with compounds or vehicle (DMSO) at the doses and times indicated. Luciferase activity in cell lysate was determined with a luciferase assay kit (Promega, Madison, WI) according to the manufacturer's instructions. Images were acquired for 1 min with a Xenogen IVIS 200 (Caliper, Hopkinton, MA). Equally sized areas were analyzed using Living Image 2.20 software.

### Clonogenic assay

Exponentially growing SiHa, CaSki and HeLa cervical cancer cells were seeded at either 1,000 or 100 cells/well in 6 well plates. One day post seeding, the cells were treated with either vehicle alone (mock) or RAMB1 at the indicated doses, and incubated at 37 C in 5% CO_2_ for 10 days. At the end of the treatment the medium was removed and the cells were rinsed with PBS prior fixation and staining of the colonies using a mixture of 6.0% glutaraldehyde and 0.5% crystal violet as previously described [27]. Colonies were imaged and counted using a Nikon Eclipse TE 2000E inverted microscope and images captured with Spot 3.5.8 acquisition software (Diagnostic Instruments, Sterling Heights, MI). All the experiments were conducted in triplicate.

### Statistical analysis

Results are reported as mean ± Standard Deviation (SD). Statistical significance of differences was assessed by two-tailed Student's *t* using Prism (V.5 Graphpad, San Diego, CA) and Excel. The level of significance was set at p≤0.05. The combination index (CI) of RAMB1 and Chloroquine was calculated by the median-effect analysis according to the method of Chou and Talaly [28]. CI<1 indicates synergism, CI = 1 indicates additivity, and CI>1 indicates antagonism. Further regression analyses were performed to stabilize estimates.

## Results

### RAMB compounds selectively reduce the viability of cervical cancer cells independently of HPV genotype *via* blockade of proteasomal degradation

Flavonoids and triterpenoids family members including celastrol [19], resveratrol [29], [30], curcumin [17], epigallocatechin-3-gallate [20], [31], [32], [33], [34] and chalcones [35], [36] exhibit anti-cancer properties associated with their activity as proteasome inhibitors [14], [15], [16], [21]. We recently reported that in chalcone-based proteasome inhibitors the nature of the aminoacid portion of the molecule confers specificity toward catalytic activities of the 20S proteasome [14]. Based upon several prior studies [22], [23], [37], we hypothesized that the α-β ketone system of chalcones may represent the minimum molecular determinant for inhibition of ubiquitin-mediated protein degradation upstream of proteasomes.

**Figure 1:**
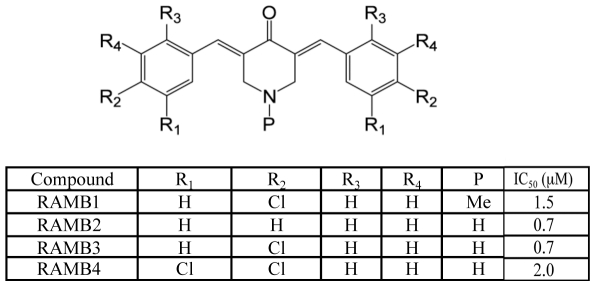
Summary of the structure of compounds RAMB1–4 and their IC_50_ values in HeLa cervical cancer cell line. IC_50_ values were determined by XXT assay. The IC_50_ value reported are average of three independent determinations.

**Figure 2:**
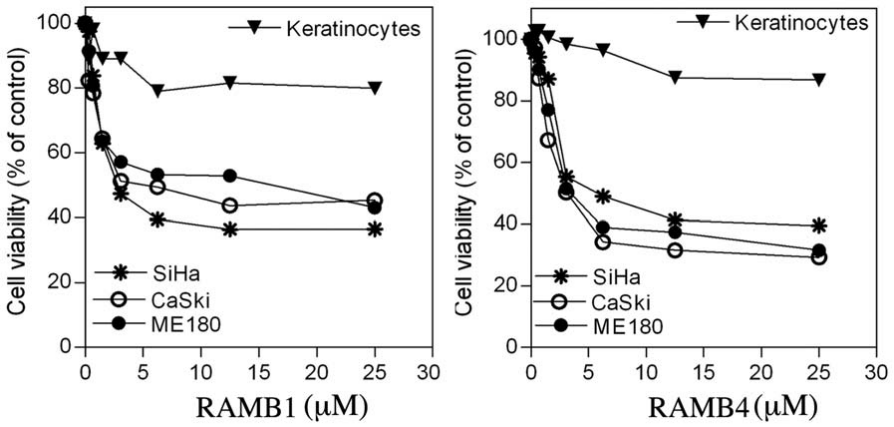
Effect of RAMB treatment upon cervical cancer cell lines and primary human keratinocytes. Cultures of HPV-transformed cervical cancer cells (SiHa, CaSki and ME180) or primary human keratinocytes were treated with the indicated concentrations of RAMB1 (*left panel*) or RAMB4 (*right panel*) over a period of 48 hours. Cell viability was determined by XTT assay and plotted as a fraction of the untreated control cultures.

**Figure 3:**
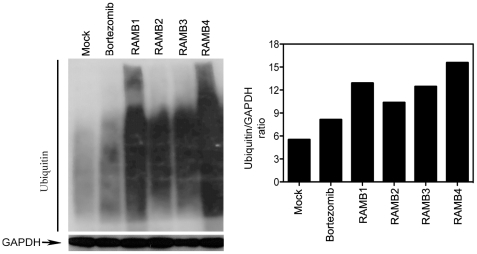
Effects of RAMB treatment on the levels of polyubiquitinated proteins in HeLa cells. *Left panel:* immunoblot analysis of ubiquitinated proteins in HeLa cells after 6 hours exposure with or without 10 µM RAMBs. Bortezomib was used as positive control. Equal protein loading in each lane was verified by using an antibody against GAPDH. *Right panel:* Quantification of the Ubiquitin/GAPDH ratios.

To test this hypothesis we initially screened a library of chalcone-based derivatives carrying various substituents on the aromatic rings adjacent to the α-β ketone system and lacking aminoacidic portions, for their cell growth inhibitory capacity in exponentially growing HPV18-positive HeLa cervical cancer cell line in a range of concentration from 100 to 0.01 µM (not shown). Four chalcone derivatives, hereafter termed RAMBs, capable of reducing the cell viability of exponentially growing HeLa cervical cancer cells in a dose-dependent fashion with IC_50_ values <5 µM, were chosen for further evaluation (Figure 1). To determine the feasibility of using RAMBs compounds for treatment of cervical cancer, we tested whether RAMB1–4 treatment would specifically hinder the cell viability of cervical cancer cells over normal keratinocytes and whether the reduction in cell viability in cervical cancer cells is dependent upon the HPV-genotype. As shown in Figure 2, RAMB1 or RAMB4 treatment produced a dose-dependent reduction in the viability of HPV16-positive SiHa and Caski cells and HPV-39-positive ME180 cervical cancer cell lines respectively with minimal effects on the viability of primary human keratinocytes and with IC_50_ similar to the obtained with HeLa. Similar results with slightly higher IC_50_ were obtained using RAMB2 and RAMB3 (not shown). To test whether the reduction in cell viability observed in cervical cancer cells following exposure to the RAMB1–4 compounds was due to their capacity of interfere with ubiquitin-mediated protein degradation, we monitored the levels of accumulation of poly-ubiquitinated proteins following treatment. Specifically, HeLa cells were treated with 5 µM of RAMB1, RAMB2, RAMB3, or RAMB4 or 10 nM of Bortezomib, the latter being used as UPS-stressor positive control, over a period of 6 hours. As shown in Figure 3 (*left panel*), immunoblot analysis of ubiquitinated protein expression levels in HeLa cells revealed a clear pattern of accumulation of poly-ubiquitinated proteins in RAMBs -treated HeLa cultures. A semi-quantitative analysis of the polyubiquitinated protein levels shows that the GAPDH-normalized levels of polyubiquitinated proteins are consistently higher (up to 3-fold) in RAMB-treated versus mock-treated cells (Figure 3, *right panel*). These findings suggest that the decrease in cell viability observed in the cervical cancer cell panel but not in normal cells following RAMBs treatment is associated with the perturbation of ubiquitin-mediated protein degradation and occurs regardless of the oncogenic HPV type.

### RAMB treatment triggers a Ubiquitin-Proteasome-System (UPS)-stress response without affecting 20S proteasome catalytic activities

**Figure 4:**
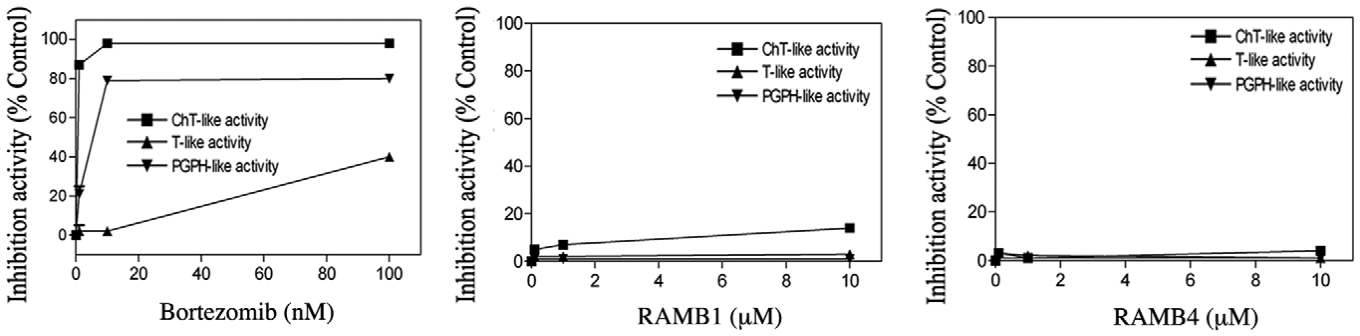
RAMB treatment fails to inhibit the 20S proteasome proteolytic activities. Purified 20S proteasomes were treated for 30 min with or without RAMB compounds or Bortezomib, here used as positive control, at the indicated concentrations and the specific fluorogenic substrates for chymotrypsin-like, trypsin-like and peptidylglutamyl peptide hydrolyzing-like hydrolytic proteasome capacities were subsequently added. A representative example of two independent experiments is shown.

To test whether the rapid (six hours or less, unpublished data) accumulation of poly-ubiquitinated proteins following RAMBs exposure occurs concomitantly with direct inhibition of the catalytic activities of the proteasomes, we tested for the ability of RAMB1 and RAMB4 (which induced a greater accumulation of polyubiquitinated protein than the other RAMBs, Figure 3) to inhibit specific catalytic sub-units within the 20S proteasome. Specifically the RAMBs were tested for their capacities to inhibit the chymotrypsin-like (CT-like), trypsin-like (T-like) and peptidylglutamyl peptide hydrolyzing-like (PGPH-like) activities in 20S purified proteasome pre-exposed to escalating doses up to 10 µM of RAMBs for a period of 30 minutes following addition of fluorogenic substrates [38]. The FDA licensed proteasome inhibitor Bortezomib was used as positive control. As shown in Figure 4 the profile of proteasome inhibition shows that unlike Bortezomib, RAMB1 and RAMB4 treatment failed to inhibit proteasomal functions when tested to concentrations up to 10 µM (similar results were obtained with the other compounds of the series, not shown).

**Figure 5:**
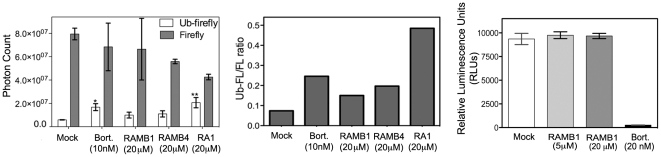
RAMB treatment fails to inhibit proteasomal activity in living cells. *Left panel:* Luciferase activity in lysates of Ub-FL or FL-vector transfected cells either with or without treatment with RAMB compounds or Bortezomib were quantified in Relative Luminescence Units (RLU) and expressed as % of control. Error bars are Standard Deviation (SD) for three independent experiments. *Middle panel:* Quantification of the Ub-FL/FL ratio. *Right panel:* Luminescence activity in cell lysates derived from CaSki cervical cancer cells with or without RAMB or Botezomib treatment quantified in Relative Luminescence Units (RLUs). *, P<0.05, **, P<0.02.

In order to assess whether the failure to inhibit proteasomal function *in vitro* can be recapitulated in the intact proteasome found in living cells, we utilized two different approaches. First, we utilized the ubiquitin-luciferase bioluminescent reporter 4XUb-FL, which resists cleavage by ubiquitin hydrolases [26], to transfect HeLa cells. Using Ub-FL and FL transfected HeLa cells we have monitored luciferase activity following exposure to RAMBs over a period of 6 hours. As shown in Figure 5 *(left)* unlike Bortezomib or our recently identified proteasome inhibitor RA1 [14], here used as proteasome inhibitors positive controls, RAMB1 or RAMB4 treatment induced a weaker stabilization of the Ub-FL reporter when tested at concentration up to 20 µM than seen for either RA-1 or Bortezomib. Quantification of Ub-FL/FL ratio in mock versus RAMBs exposed culture is provided in Figure 5 (*middle* panel). Next, the lack of inhibition of 20S proteasomal activity in RAMB-treated cells was confirmed by measuring the residual fluorogenic activity in 20S proteasome purified from CaSki cervical cancer cells pre-exposed to RAMB1 or RAMB4 for 4 hours. As shown in Figure 5 (*right panel*), unlike Bortezomib, RAMB1 treatment failed to inhibit the chymotryptic activity of proteasomes when tested to concentrations up to 20 µM. Taken together, this suggests that the loss of cell viability in cervical cancer cells following RAMBs exposure occurs concomitantly with the accumulation of polyubiquitated proteins without the direct inhibition of 20S proteasomal activity.

### RAMB1 treatment induces aggresome formation and triggers heat-shock responses

**Figure 6:**
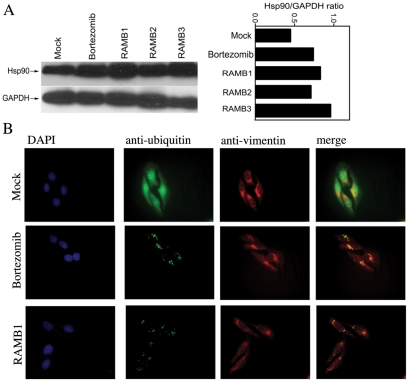
RAMB treatment induces accumulation of Hsp90 and aggresomes. A. *Left panel:* immunoblot analysis of Hsp90 expression levels in HeLa cervical cancer cells after 8 hours exposure with or without 10 µM RAMBs treatment. Bortezomib was used as positive control. Equal protein loading in each lane was verified by using an antibody against GAPDH. *Right panel:* Quantification of the Hsp90/GAPDH ratio. B. HeLa cells were incubated with or without 5 µM RAMB1 or 10 nM Bortezomib for 18 hours before fixation and immuno-fluorescent staining of DNA (blue), ubiquitin (green) and vimentin (red) before imaging (60×).

We and others have shown that inhibition of ubiquitin-mediated protein degradation via proteasomal inhibition triggers heat-shock and unfolded protein responses including formation of cytoprotective structures called aggresomes as a mechanism to compensate for inhibition of proteasomal functions and increasing levels of UPS stress within cancer cells [4], [6], [39]. To test whether the rapid accumulation of poly-ubiquitinated protein upon RAMB1 treatment occurs concomitantly with heat-shock protein responses (UPR) we monitored the protein expression levels of Hsp90 in HeLa cervical cancer cells exposed to 10 µM RAMB1–3 for 8 hours. As shown in Figure 6A (*left panel*) immunoblot analysis of Hsp90 protein expression levels revealed that a strong pattern of accumulation of Hsp90 in RAMB1-treated versus mock-treated HeLa cell cultures (similar results were obtained with the other derivative of the series, not shown). A semi quantitative analysis of the Hsp90 protein levels show that the GAPDH-normalized levels of polyubiquitinated proteins are nearly 2-fold higher in treated versus non-treated cells is shown in Figure 6A (*right panel*).

Next, we hypothesized that accumulation of poly-ubiquitinated proteins following RAMB compound exposure would lead to activation of alternative compensatory pathways to ubiquitin-mediated protein degradation, specifically to lysosomal pathway activation. To test this hypothesis we monitored the sub-cellular localization of ubiquitin by immunofluorescence microscopy analysis in HeLa cervical cancer cells exposed to 10 µM of RAMB1. As shown in Figure 6B immunofluorescence analysis of poly-ubiquitinated proteins in HeLa cells treated with RAMB1 reveals the presence of vimentin-caged, ubiquitin-positive, aggresomes structure consistent with that previously described upon treatment with Bortezomib [6]. Taken together, these findings indicate that in RAMB1-treated cells the accumulation of poly-ubiquitinated results in similar cytoprotective responses as inhibition of proteasome catalytic activities by Bortezomib, but occurs through a mechanism independent from it.

### RAMB1 treatment leads to p53 stabilization, cyclin D1 destabilization and onset of apoptosis

**Figure 7:**
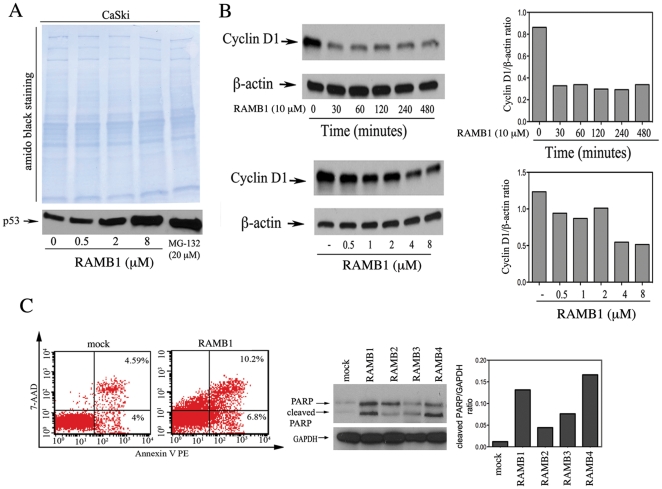
Stabilization of p53, reduction in cyclin D1 and onset of apoptosis following RAMB1 treatment. A. Immunoblot analysis of p53 expression level in CaSki cells treated with the indicated concentration of RAMB1 over a period of 8 hours. Equal loading is each lane was verified by amido black staining. B. *Top left panel:* Immunoblot analysis of cyclin D1 expression level in CaSki cells with or without 10 µM RAMB1 for a period up to 8 hours. Protein loading was examined using an antibody against β-actin. *Top right panel:* Quantification of the cyclin D1/β-actin ratio. *Bottom left panel:* Immunoblot analysis of cyclin D1 expression level in CaSki cells with or without RAMB1, at the indicated concentration for a period of 8 hours. Equal protein loading in each lane was verified by using an antibody against β-actin. *Bottom right panel:* Quantification of the cyclin D1/β-actin ratio. C. *Left panel*. HeLa cells were treated with 10 µM of RAMB1 for 8 hours and analyzed by flow cytometry after staining for annexin V binding and 7-AAD incorporation. *Middle panel*: Immunoblot analysis of full length and cleaved PARP in HeLa cells treated 10 µM of RAMBs over a period of 8 hours. Protein loading was assessed using an antibody against β-actin. *Right panel*: Quantification of cleaved PARP/β-actin ratio.

The E6 oncoprotein of HPV exerts its oncogenic activity by targeting p53 and other tumor suppressor proteins for rapid ubiquitin-mediated proteasomal degradation. This reduces the level of this key cellular cell cycle regulator without mutation of p53. To test whether the impairment of ubiquitin-mediated protein degradation following RAMBs treatment leads to stabilization of p53 as a potentially contributing mechanism initiating cell death, we examined the expression levels of p53 following 6 hours exposure to compounds 10 µM of RAMBs. As shown in Figure 7A, 8 hours RAMB1 exposure is associated with dose-dependent accumulation of p53 in CaSki cervical cancer cells as compared to mock control. Importantly, p53 stabilization causes suppression of cyclin D1 promoter resulting in active repression of cyclin D1 transcription [40]. To test whether this results in reduction of cyclin D1 expression levels, CaSki cervical cancer cells were exposed to increasing doses of RAMB1 over a period up to 8 hours. As shown in Figure 7B *(left panel)* RAMB1 treatment causes time-dependent (*top*) and dose-dependent (*bottom*) decrease of cyclin D1 levels suggesting failure of cervical cancer to enter the S- phase of the cell cycle as a cause of cell toxicity [41]. A semi quantitative analysis of β-actin-normalized cyclin D1 levels is given in Figure 7B (*right panel top and bottom*).

To test whether this results in reduction of cyclin D1 expression levels, CaSki cervical cancer cells were exposed to increasing doses of RAMB1 over a period up to 8 hours. A semi quantitative analysis of β-actin-normalized cyclin D1 levels is given in Figure 7B (*right panel top and bottom*). As shown in Figure 7B *(left panel)* RAMB1 treatment causes very rapid (*top*) and dose-dependent (*bottom*) decrease of cyclin D1 levels suggesting failure of cervical cancer to enter the S-phase of the cell cycle may contribute to the loss of cell viability [41].

Stabilization of p53 steady-state levels and destabilization of cyclin D1 levels suggest that the reduction in cell viability observed in the panel of cervical cancer cells following RAMBs exposure might trigger the onset of apoptosis. To test this hypothesis, HeLa cervical cancer cells were exposed to 5 µM of RAMB1 and analyzed by flow cytometry after staining for annexin V binding and 7-AAD incorporation. Annexin V and 7-AAD staining allows to discriminate viable (annexin V^neg^/7-AAD^neg^), apoptotic (annexin V^pos^/7-AAD^neg^), and dead (annexin V^pos^/7-AAD^pos^) cells. As shown in Figure 7C (*left panel*), 24 hr exposure to RAMB1 caused an increase in both the early (annexin V positive population) and late (annexin V and 7-AAD double positive population) and apoptotic population in treated cultures versus controls. To evaluate whether this is due to caspase activation, we measured the levels of PARP cleavage in HeLa cancer cells following exposure to RAMB1. As shown in Figure 7C (*middle panel*), higher levels of cleaved PARP are apparent in treated versus untreated cultures indicating caspase-3 activation. Quantification of the cleaved PARP/GAPDH is provided in Figure 7C (*right panel*). These findings support our hypothesis that RAMBs treatment triggers apoptosis in cervical cancer cells.

### RAMB1 treatment prevents anchorage-dependent tumor-colony formation of cervical cancer cells and synergizes with the lysosome inhibitor Chloroquine

**Figure 8:**
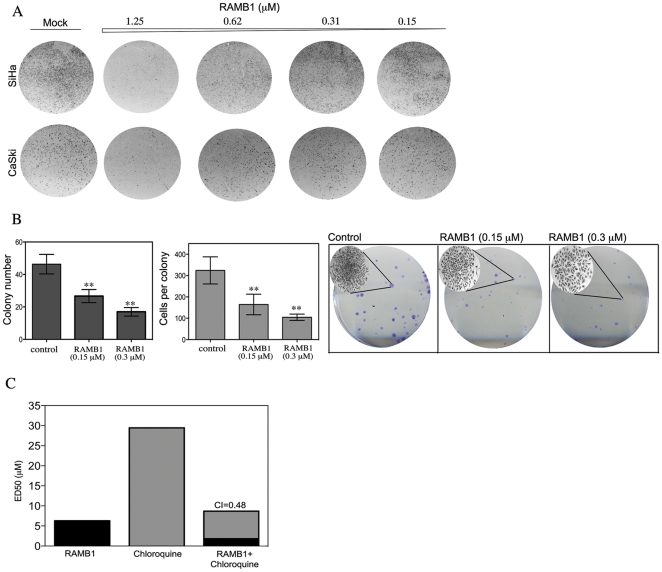
RAMB1 treatment prevents anchorage-dependent colony formation and synergizes with Chloroquine to kill cervical cancer cells. A. Equal numbers of SiHa and Caski cells (10^3^) were seeded into 6-wells plastic dishes and treated with or without RAMB1 at the indicated concentrations over a period of 10 days. Colonies were visualized by crystal violet staining. B. *Left panel*: Quantification of colony number in mock versus RAMB1 treated HeLa cells. *Middle panel*: Quantification of colony size in mock versus RAMB1 treated cervical cancer cells. *Right panel*: Representative experiment of HeLa cells (10^2^) which were seeded into 6-wells plastic dishes and treated with or without RAMB1 at the indicated concentration over a period of 10 days. Colonies were visualized by crystal violet staining and manually counted using an inverted microscope. *Inserts:* Representative example of colony size in mock versus RAMB1-treated cells. *Middle panel:* Quantification of colony number. *Right panel*: Quantification of colony size, representative of three independent experiments. **, P<0.02. C. CaSki cells were treated with checkerboard dilution series of RAMB1 and Chloroquine over a period of 24 hours. Cell viability was measured by XTT assay and calculated as percent of control untreated cultures. Synergy is expressed as combination index (CI).

A hallmark of cancer cells is their capacity to form colonies *in vitro* that is indicative of the loss of contact inhibition typical of normal cells. To test the potential of RAMB1 as a therapeutic agent for cervical cancer, we have examined the effects of RAMB1 treatment on the anchorage-dependent growth of SiHa, CaSki and HeLa cervical cancer cell lines. Specifically, SiHa and CaSki cancer cells were either mock treated or treated with escalating doses of RAMB1 over a period of 10 days and their ability to form colonies was evaluated by crystal violet staining. As shown in Figure 8A, RAMB1 exposure resulted in a dose-dependent inhibition of colony formation by SiHa and CaSki cervical cancer cells as compared to mock-treated cultures when used at concentrations as low as 0.62 µM.

We next quantified the impact of RAMB1 treatment on the reduction of colony number and size in 1×10^2^ HeLa cervical cancer cells treated with or without 0.15 or 0.5 µM RAMB1. As shown in Figure 8B (*left panel*) RAMB1 exposure resulted in 40% and 60% reduction in colony number when exposed to 0.15 or 0.3 µM RAMB1 respectively. RAMB1 treatment also resulted in 50% and 80% reduction in colony size in mock versus RAMB1 treated HeLa cells as shown in Figure 8B (*middle panel*). A representative example of crystal violet staining in HeLa cervical cancer cells following RAMB1 exposure is given in Figure 8B (*right panel*).

The increase in the steady-levels of Hsp90 and formation of aggresome-like structure following RAMB1 treatment suggest compensatory activation of lysosomal pathway of protein degradation (Fig. 6). Therefore, we hypothesized that RAMB1 treatment would result in synergistic cell killing when combined with an inhibitor of this lysosomal pathway. To test this hypothesis, we compared the effect of combined treatment with RAMB1 and the lysosome inhibitor Chloroquine on the viability of HeLa cervical cancer cells. Consistent with our hypothesis, submaximal doses of RAMB1 and Chloroquine act synergistically to cause enhanced cytotoxicity in HeLa cervical cancer cells with a Combination Index (CI) of 0.48 observed using 1.45 µM RAMB1 and 7.23 µM Chloroquine (Figure 7C). Similar results were obtained with SiHa and CaSki cell lines (data not shown). Taken together, this suggest that the combinatorial approach of inhibiting ubiquitin-dependent protein degradation upstream of proteasome and lysosomal pathway could lead to development of combination therapeutic strategies where the side effects of each individual compounds can be reduced without compromising the anti-cancer activity of the treatments.

## Discussion

Numerous naturally-derived compounds are either FDA approved or currently under evaluation for the treatment of human malignances. Among these natural compounds with promising anticancer properties are members of the flavonoid and triterpene families. While the chemical common denominator within these two families capable of inducing preferential killing in cancer cells is yet to be identified, a number of studies support the idea that they share an ability to inhibit one or multiple catalytic activities of the proteasome, and that their anti-cancer activities stems from this inhibition [14], [15], [17], [21].

While we have recently shown that in chalcone-based small molecule inhibitors of proteasome the nature of aminoacidic component determines the selectivity toward individual catalytic activities of the proteasome [14], a substantial body of evidence indicates that chalcones may have within their β-carbons to α,β-unsaturated carbonyls the molecular determinant for inhibition of ubiquitin-dependent protein degradation upstream of proteasomes [22], [23], [24], [25]. Thus, they may exert anticancer activity by induction of UPS stress via a mechanism independent from direct inhibition of the catalytic activities of the 20S proteasome.

Given these observations and based on our recent work [14], we have synthesized a library of chalcone-based small molecules (RAMBs) containing α,β-unsaturated carbonyl systems and lacking aminoacid substitutions and tested their antineoplastic potential *via* inhibition of ubiquitin-mediated protein degradation upstream of proteasomes in the cervical cancer setting. Our results show that, when probed for their capacity to interfere with degradation of poly-ubiquitinated proteins, RAMB treatment is associated with increased levels of poly-ubiquitinated proteins (occurring as early as two hours from exposure) but unaltered 20S proteasomal catalytic activity when tested *in vitro* and in living cells. Notably, while not statistically significant, RAMB1 treatment led to some stabilization of the Ub-FL degron. This may reflect a feedback inhibition of proteasome function due to increasing levels of poly-ubiquitinated protein content in RAMB1 exposed cultures.

Importantly, when tested against a panel of cervical cancer cell lines versus keratinocytes, RAMBs showed a preferential killing for the malignant versus the normal phenotype. This appears to be consistent with the notion that due to their endogenously high levels of UPS stress cancer cells are more sensitive than normal cells to perturbation of ubiquitin-mediated protein degradation [4].

Activation of p53 via its stabilization is a potential therapeutic approach for treatment of those cancers, including cervical cancer, expressing wild-type p53. E6 oncoprotein transforms cervical cancer cells by targeting p53 for ubiquitin-dependent proteasomal degradation resulting in reduction of the levels of this and other tumor-suppressor proteins. Therefore, stabilization of p53 by preventing its degradation could recover sufficient levels of wild-type p53 to trigger apoptotic cell death as a response to the abnormal growth of the cancer cell. We show here that RAMB1 treatment causes dose-dependent accumulation/stabilization of p53. Because stabilization of the levels of p53 occurs concomitantly with accumulation of high-molecular weight poly-ubiquitinated species and as early as 2 hours post-treatment (well before cell death is observed, not shown), this suggests that stabilization of p53 is the cause rather then the consequence of the decrease in cell viability in cervical cancer cells. Induction of p53 via stabilization of its steady state levels correlates with decrease in the levels of cyclin D1 possibly via repression of its transcription [40]. RAMB1 treatment also results in a dose- and time-dependent reduction in the steady state levels of cyclin D1 which could at least partially account for the decrease in cell viability observed in the cervical cancer cell lines. Interestingly, because the decrease in the cyclin D1 expression levels are seen as early as 30 minutes following RAMB1 exposure and before detectable stabilization of p53 expression levels, it is possible that cyclin D1 down-regulation is occurring in response to UPS stress rather to p53 activity. In this scenario an arrest in S-phase would precede the onset of apoptosis in RAMBs exposed cervical cancer cells as confirmed by Annexin V staining and PARP-cleavage. This is likely the same mechanism responsible for reduction in colony number observed in the panel of cervical cancer cell lines exposed to RAMB1 treatment. Interestingly, because the reduction in colony number is accompanied by reduction in colony size we conclude that RAMB1 has an effect in slowing the proliferation rate of cervical cancer cells that are initially resistant to its action.

Inhibition of conventional (proteasomal-mediated) ubiquitin-dependent protein degradation triggers activation of alternative pathways to proteasomal degradation as a mechanism to compensate for abnormally increased levels of UPS stress. The increase in the levels of poly-ubiquitinated proteins following RAMB1 exposure corresponds to their sub-cellular localization in vimentin-encaged structures consistent with the formation of aggresomes and occurs concomitantly with up-regulation of Hsp90 protein expression levels. This indicates activation of the lysosomal protein degradation pathway as an attempt to “isolate” mis-folded and oxidized proteins and subsequent recycling of the aggresomes via autophagy to cope with increasing levels of UPS stress [6], [39]. In this scenario we show that a combinatorial approach of using RAMB1 and the lysosome inhibitor Chloroquine reduce the viability of cervical cancer cells significantly better than either treatment alone. This synergism presumably reflects blockade of the proteasomal and compensatory lysosomal degradation pathways by RAMB1 and Chloroquine respectively, and provides a rational to further explore this new therapeutic approach for the treatment of cervical cancer.
